# Correlation between Cytotoxic Activities and Reduction Potentials of Heterocyclic Quinones

**DOI:** 10.3390/molecules15096559

**Published:** 2010-09-20

**Authors:** Junko Koyama, Izumi Morita, Takao Yamori

**Affiliations:** 1 Faculty of Pharmaceutical Sciences, Kobe Pharmaceutical University, Higashinada, Kobe 658-8558, Japan; E-Mail: t-izumi@kobepharma-u.ac.jp (I.M); 2 Division of Molecular Pharmacology, Cancer Chemotherapy Center, Japanese Foundation for Cancer Research, Tokyo, Japan; E-Mail: yamori@jfcr.or.jp (T.Y.)

**Keywords:** human cancer cell lines, heterocyclic quinone, reduction potential, cyclic voltammetry

## Abstract

To search for possible anti-tumor agents or anti-tumor promoters among natural or synthetic products, we used cyclic voltammetry to determine the reduction-oxidation potentials of heterocyclic quinones in phosphate buffer at pH 7.2. We determined the growth inhibitory- and cytotoxic activities of 12 heterocyclic quinone anti-tumor agent candidates against a panel of 39 human cancer cell lines (JFCR39). The average concentrations of the heterocyclic quinones required for 50% growth inhibition (GI_50_) against JFCR39 ranged from 0.045 to 13.2 μM, and the 50% lethal concentration (LC_50_) against JFCR39 ranged from 0.398 to 77.7 μM. The average values of GI_50_ or LC_50_ of the heterocyclic quinones correlated significantly with their reduction potentials. These results suggested that reduction-oxidation potentials could be a useful method for the discovery of novel antitumor agents.

## 1. Introduction

Naturally occurring quinones, which are widely distributed throughout both the animal and plant kingdoms, typically function as pigments and as intermediates in cellular respiration and photosynthesis. Several of these molecules possess anti-neoplastic chemotherapeutic properties [1,2]. Quinones are found in many drugs, including anthracyclines, daunorubicin, doxorubicin, mitomycin, mitoxantrones, and saintopin, all of which are used in the clinical therapy of solid tumors. The cytotoxic effects of these quinones are primarily due to inhibition of DNA topoisomerase-II [3,4]. Several recent publications have highlighted the anti-tumor activity of kigelinone (2-(1-hydroxyethyl)-5(or 8)-hydroxynaphtho[2,3-*b*]furan-4,9-dione), a furanonaphthoquinone molecule isolated from *Tabebuia cassinoide*s [5]. Heterocyclic quinones containing nitrogen atoms possess excellent anti-tumor [6,7] and other biologic activities [8,9]. Previously, we reported the *in vitro* anti-tumor promoting activity of heterocyclic quinines, as evidenced by inhibitory effects on 12-*O*-tetradecanoylphorbol-13-acetate (TPA)-induced Epstein-Barr virus early antigen (EBV-EA) activation in Raji cells [10,11]. Standard redox potential is important in determining the physiological activity of drugs [12]. We employed cyclic voltammetry to determine the standard redox or first reduction potentials of anthraquinones, bianthraquinones, naphthoquinones, and azaanthraquinones at a physiological pH of 7.2. We found a significant correlation between the standard redox or first reduction potentials and the inhibitory effects (log IC_50_) of these compounds on EBV-EA activation [13,14,15,16,17,18]. 

In this study, we have attempted to expand the potential use of reduction-oxidation potentials determined by cyclic voltammetry to the discovery of anti-tumor agents. We determined the growth inhibitory- and cytotoxic activities of 12 heterocyclic quinine anti-tumor agent candidates against a panel of 39 human cancer cell lines (JFCR39), an information-rich and pharmacologically well characterized drug discovery system [19,20,21,22,23]. Subsequently, we measured the reduction-oxidation potentials of these compounds in phosphate buffer at pH 7.2. Then, we examined the correlation between the activities (log GI_50_ and log LC_50_) and first reduction potentials of these compounds. Furthermore, we calculated additional molecular properties of heterocyclic quinones using the CAChe MOPAC program and the PM3 method [24], and identified the partition coefficient (log *P*) and LUMO energy as useful parameters that could help predict anti-tumor activities of these compounds.

## 2. Results and Discussion

**Figure 1:**
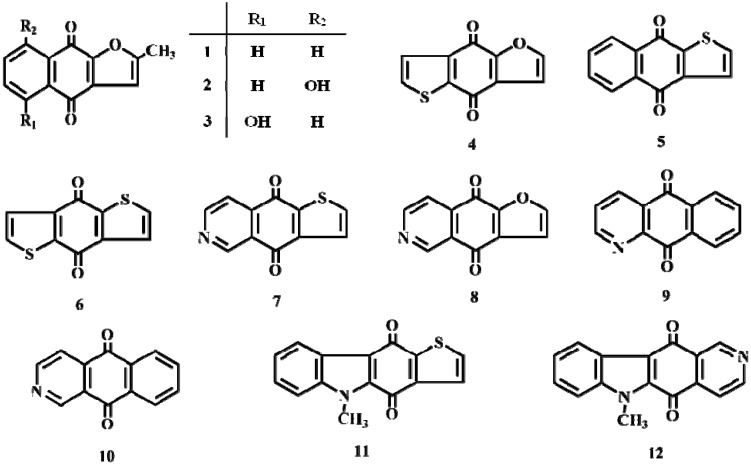
Structures of heterocyclic quinone derivatives.

Twelve heterocyclic quinones (Figure 1) were tested for their growth inhibitory- and cytotoxic activities against JFCR 39 cells; the results (the means of GI_50_ and LC_50_ values) are summarized in Table 1. Compounds **2**-**4**, **6**-**8**, and **12** were cytotoxic (GI_50_: 0.045-0.831 μM). Compounds **7** and **8** exhibited the highest levels of cytotoxicity, with GI_50_ values of 0.071 and 0.045 μM and LC_50_ values of 0.724 and 0.398 μM, respectively. In contrast, compounds **9** and **11** were minimally cytotoxic, with GI_50_ values of 13.18 and 9.33 μM, respectively. The presence of a phenolic hydroxy group increased the potency of compounds having similar backbone molecular structures (**1→2**,**3**); a similar tendency was observed for anthraquinones [18]. Replacement of the thiophene for a furan ring showed a similar tendency in activity (**4** and **6**, **7** and **8**). The pyridine ring with the nitrogen in the 2 position lead to heteroquinones with good potency. Replacement of the pyridine (2 position of the nitrogen) for a thiophene ring reduced the activity (**7**>**6**, **8**>**4**, **12**>**11**). 

**Figure 2:**
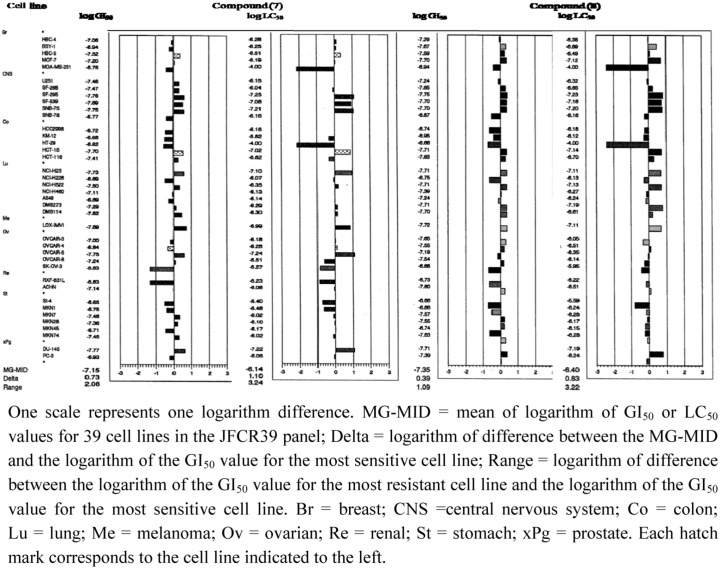
Growth inhibitory- and cytotoxic activities of compounds **7** and **8** against 39 human cancercell lines in the JFCR39 panel. Mean graph was produced by computer processing of the 50% growth inhibition (GI_50_) values. Logarithm of the GI_50_ value for each cell line is indicated. In the plot, columns to the right of zero indicate that thesensitivity of the cell line to the compound, and columns to the left indicate resistance to the compound. The x–axis represents logarithm of difference between the mean of GI_50_ valuesfor 39 cell lines and the GI_50_ value for each cell line in the JFCR39 panel.

The graphs displaying the means of the growth-inhibition of GI_50_ and cytotoxicity (LC_50_) of compounds (**7** and **8**) demonstrated the potent anti-cancer effects of these compounds (Figure 2). The GI_50_ and LC_50_ values for compounds **7** and **8** indicated that these compounds were more effective against the lung cancer line NCI-H23, the colorectal cancer cell line HCT-15, the melanoma line LOX-IMVI, the central nervous system cell lines SF-295 and SNB-75, and the prostate cancer line DU-145.

**Table 1:** Growth inhibitory- (GI_50_) and cytotoxic (LC_50_) activities of heterocyclic quinone derivatives against 39 human cancer cell lines in the JFCR39 panel.

	MG-MID of log GI_50_	(μM)	MG-MID of log LC_50_	(μM)
**1**	-5.56	(2.75)	-5.04	(9.12)
**2**	-6.08	(0.831)	-4.84	(14.45)
**3**	-6.29	(0.513)	-5.18	(6.61)
**4**	-6.52	(0.302)	-5.83	(1.48)
**5**	-5.38	(4.17)	-4.26	(54.95)
**6**	-6.54	(0.288)	-5.70	(2.00)
**7**	-7.15	(0.071)	-6.14	(0.724)
**8**	-7.35	(0.045)	-6.40	(0.398)
**9**	-4.88	(13.18)	-4.11	(77.62)
**10**	-5.69	(2.04)	-4.63	(23.44)
**11**	-5.03	(9.33)	-4.13	(74.13)
**12**	-6.12	(0.759)	-5.03	(9.33)

GI_50_: 50% Growth inhibition concentration (M); LC_50_: 50% Lethal concentration (M); MG-MID: Mean of logarithm of GI_50_ or LC_50_ values for 39 cell lines in the JFCR39 panel.

The COMPARE algorithm was established for the JFCR39 panel, which can predict the mode of action of a test compound based on the similarity in the fingerprint (see Figure 2), a growth inhibition profile of a compound across the JFCR-39 panel, to the reference compounds with known mode of actions [19,21]. We analyzed the fingerprints of the heterocyclic quinone derivatives. In the development of the heterocyclic quinones, furanonaphthoquinones went ahead, and, as for **1**, anti-tumor activity was already proved *in vivo* [25]. Compounds **4** and **6** were designed based on compound **1**, and compounds **7** and **8** were designed based on **4**. Compounds **7** and **8** were apparently more cytotoxic *in vitro* than **4.** Although compounds **4** and **6-8** inherited a characteristic of compound **1,** they were different in the fingerprints from any of known anti-cancer drugs listed in the database of the JFCR-39 [20], suggesting that these compounds had very unique modes of action (data not shown). We are interested in whether these compounds might display anti-tumor effects *in vivo*.

**Figure 3:**
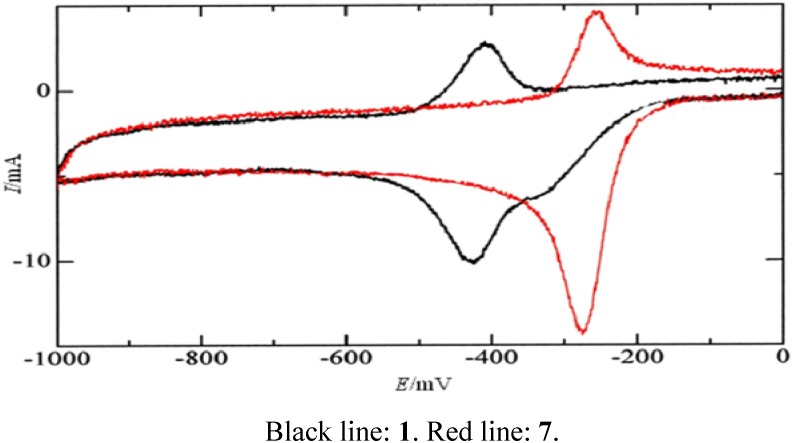
Cyclic voltammograms of compounds **1** and **7** at a PFC electrode in 3:1 (v/v) 0.1 M phosphate buffer (pH 7.2) – ethanol. Voltage scan rate: 20 mV s^-1^.

**Figure 4:**
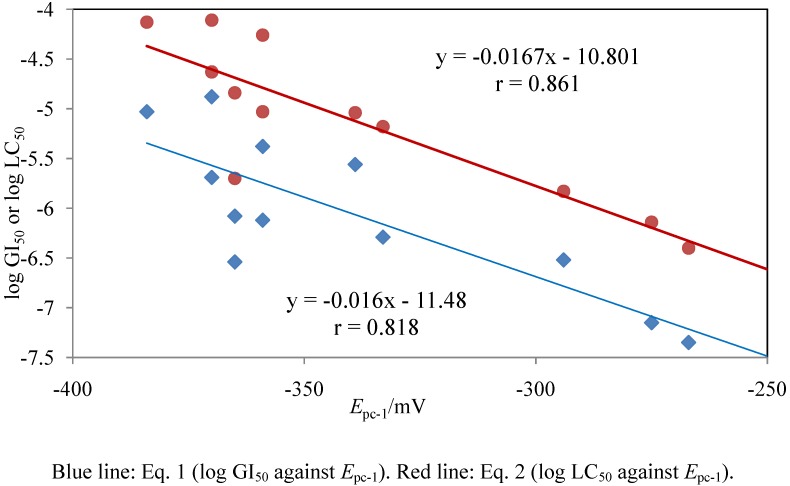
Regression plot of log GI_50_ or log LC_50_ and the first reduction potential at pH 7.2 of heterocyclic quinone derivatives with their cytotoxic activity.

Cyclic voltammograms of **1** and **7** obtained at 20 mV s^–1^ (Figure 3) demonstrated that compound **1** produced two (one is a shoulder peak) cathodic (reduction) peaks, while compound **7** produced one cathodic peak (cathodic and anodic peak potentials at 20 mV s^–1^ are summarized in Table 2). When the voltage scan rate was increased (up to 100 mV s^–1^), the peak current of the second sharp reduction peak (accompanied by an anodic peak) increased, to become larger than that of the first reduction peak; therefore, this peak pair was an adsorption wave due to the redox reaction of the heterocyclic quinone derivative adsorbed at the electrode surface. The corresponding anodic peak, however, was not detected for all compounds tested, likely due to the instability of the reduction product. Accordingly, the first reduction peak potential at 20 mV s^–1^ (*E*_pc-1_) was used to examine any connection to cytotoxic activity. The values of log GI_50_ or log LC_50_ were plotted against the first reduction potential (*E*_pc-1_ in mV) of compounds **1**-**12** in Figure 4. Plots demonstrated a correlation of the log GI_50_ or log LC_50_ values with *E*_pc-1_. The log GI_50_ and log LC_50_ were represented by the regression equations:

log GI_50_ = –0.016 *E*_pc-1_ – 11.48 (*n* = 12, *r* = 0.818) (1)

log LC_50_ = –0.0167 *E*_pc-1_ – 10.801 (*n* = 12, *r* = 0.861) (2)

where *n* and *r* are the numbers of test compounds and correlation coefficients, respectively.

In studies examining structure-activity relationships, electronic properties have typically been used as useful parameters. We therefore examined the correlation of log GI_50_ and log LC_50_ with the electronic properties of heterocyclic quinones (**1**-**12**), including HOMO, LUMO energy, steric energy, total energy, solvent accessible surface area (SASA), and log *P* (Table 3). Log *P* was determined to be the most effective parameter:

log GI_50_ = –6.732 + 0.807 log *P* (*n* = 12, *r* = 0.789) (3)

log LC_50_ = –5.814 + 0.835 log *P* (*n* = 12, *r* = 0.820) (4)

LUMO energy also correlated well with anti-tumor activity:

log GI_50_ = 0.822 + 4.075 LUMO (*n* = 12, *r* = 0.730) (5)

log LC_50_ = 1.453 + 3.891 LUMO (*n* = 12, *r* = 0.700) (6)

We performed multiple regression analyses for the log GI_50_ and log LC_50_ values using electronic and molecular properties of the heterocyclic quinone derivatives:

log GI_50_ = –6.097 – 0.012 *E*_pc-1_ – 2.338 LUMO (*n* = 12, *r* = 0.893) (7)

log GI_50_ = –9.864 – 0.010 *E*_pc-1_ + 0.429 log *P* (*n* = 12, *r* = 0.869) (8)

log LC_50_ = –6.357 – 0.013 *E*_pc-1_ – 1.930 LUMO (*n* = 12, *r* = 0.911) (9)

log GI_50_ = –9.187 – 0.011 *E*_pc-1_ + 0.428 log *P* (*n* = 12, *r* = 0.910) (10)

Thus, *E*_pc-1_, LUMO, and log *P* were promising parameters to predict GI_50_ and LC_50_. It remains unclear, however, why these parameters correlate well with the GI_50_ and LC_50_ values of hererocyclic quinones. 

**Table 2:** First and second cathodic peak potentials (*E*_pc-1_ and *E*_pc-2_) and the anodic peak potential (*E*_pa_) versus Ag/AgCl (saturated NaCl) obtained at 20 mVs^-1^ for heterocyclic quinone derivatives.

Compound	*E*_pc-1_ (mV)	*E*_pc-2_ (mV)	*E*_pa_ (mV)
**1**	- 339	- 426	- 400
**2**	- 365	- 435	- 417
**3**	- 333	- 429	- 411
**4**	- 294	–	- 282
**5**	- 359	- 245	- 431
**6**	- 365	–	- 330
**7**	- 275	–	- 253
**8**	- 267	–	- 245
**9**	- 370	- 245	- 374
**10**	- 370	–	- 362
**11**	- 384	–	–
**12**	- 359	(- 640)	- 336

**Table 3:** Electronic properties of heterocyclic quinone derivatives.

	Steric energy (kcal/mole)	Total energy (eV)	LUMO (eV)	HOMO (eV)	SASA*^3^	log *P*
**1**	-11.449	-114.15	-1.470	-9.626	102.26	1.750
**2**	-16.057	-126.4	-1.387	-9.523	104.58	1.466
**3**	-16.000	-126.4	-1.407	-9.545	104.52	1.466
**4**	-0.746	-103.66	-1.809	-9.974	94.668	-0.230
**5**	-13.257	-104.02	-1.685	-9.994	100.34	1.286
**6**	-3.978	-100.72	-1.914	-10.064	100.42	0.113
**7**	-10.420	-106.18	-1.913	-10.148	99.581	-0.026
**8**	-6.835	-109.12	-1.774	-10.087	93.707	-0.369
**9**	-17.281	-109.49	-1.537	-10.258	99.896	1.547
**10**	-19.072	-109.49	-1.636	-10.339	99.362	1.148
**11**	-5.210	-131.74	-1.647	-8.749	118.07	1.070
**12**	-11.186	-137.2	-1.631	-8.835	116.86	0.931
r*^1^	0.396	0.247	0.730	0.250	0.432	0.789
r*^2^	0.508	0.346	0.700	0.290	0.498	0.820

r*^1^: Correlation coefficient with log GI_50_; r*^2^: Correlation coefficient with log LC_50_; r*^3^: Solvent accessible surface area.

## 3. Experimental

### 3.1. Instruments, reagents and materials

List analytical instruments used-MS and NMR data are given. 2-Methylnaphtho[2,3-*b*]furan-4,9-dione (**1**), 2-methyl-8-hydroxynaphtho[2,3-*b*]furan-4,9-dione (**2**), and 2-methyl-5-hydroxynaphtho[2,3-*b*]furan-4,9-dione (**3**) were synthesized from 2-acetyl-5-methyl-furan and phthalic anhydride derivatives [25]. Thieno[2,3-*b*]benzofuran-4,8-dione (**4**), naphtho[2,3-*b*]thiophen-4,9-dione (**5**), benzo[1,2-*b*:4,5-*b*’]dithiophene-4,8-dione (**6**), thieno[2,3-*g*]isoquinoline-4,9-dione (**7**), furano[2,3-*g*]isoquinoline-4,9-dione (**8**), 5-methyl-4H-thieno[3,2-*b*]carbazole-4,10(5H)-dione (**11**), and 6-methyl-5H-pyrido[4,3-*b*]-carbazole-5,11(6H)-dione (**12**) were prepared by tandem-directed metalation reaction [26]. Benzo[*g*]quinoline-5,10-dione (**9**) was synthesized from 5,8-quinolone and cyclohexadiene derivatives [11]. Benzo[*g*]isoquinoline-5,10-dione (**10**) was synthesized from 5,8-isoquinolone and cyclohexadiene derivatives [27].

*Furano[2,3-g]isoquinoline-4,9-dione* (**8**). HR-EI-MS *m/z*: 199.0281 (Calcd. for C_11_H_5_NO_3_, 199.0269); ^1^H-NMR (300 MHz, CDCl_3_) δ: 7.05 (1H, d, *J* = 1.8 Hz, 2-H), 7.87 (1H, d, *J* = 1.8 Hz, 3-H), 8.03 (1H, d, *J* = 5.0 Hz, 8-H), 9.10 (1H, d, *J* = 5.0 Hz, 7-H), 9.43 (1H, s, 5-H).

*5-Methyl-4H-thieno[3,2-b]carbazole-4,10(5H)-dione* (**11**). HR-EI-MS *m/z*: 267.0349 (Calcd. for C_15_H_9_NO_2_S, 267.0353); ^1^H-NMR (300 MHz, CDCl_3_) δ: 4.19 (3H, s, CH_3_), 7.41 (3H, m, 3,6,9-H), 7.55 (2H, m, 7,8-H), 8.34 (1H, d, *J* = 5.0 Hz, 2-H). 

### 3.2. Cell lines and cell cultures

The panel of human cancer cell lines, described by Yamori *et al.* [19,20,21,22,23], consists of the following 39 human cancer cell lines: lung cancer, NCI-H23, NCI-H226, NCI-H522, NCI-H460, A549, DMS273, and DMS114; colorectal cancer, HCC-2998, KM-12, HT-29, HCT-15, and HCT-116; gastric cancer, MKN-1, MKN-7, MKN-28, MKN-45, MKN-74, and St-4; ovarian cancer, OVCAR-3, OVCAR-4, OVCAR-5, OVCAR-8, and SK-OV-3; breast cancer, BSY-1, HBC-4, HBC-5, MDA-MB-231, and MCF-7; renal cancer, RXF-631L and ACHN; melanoma, LOX-IMVI; glioma, U251, SF-268, SF-295, SF-539 , SNB-75, and SNB-78; and prostate cancer, DU-145 and PC-3. All cell lines were cultured at 37 ºC under 5% CO_2_ in RPMI 1640 medium (Nissui Pharmaceutical, Tokyo, Japan) supplemented with 5% fetal bovine serum, penicillin (100 units/mL), and streptomycin (100 μg/mL).

Inhibition experiments were performed to assess the sensitivity of cells to various chemicals as described by Yamori *et al*. [19]. Growth inhibition was assessed using a sulforhodamine B (SRB) assay to determine the changes in total cellular protein after cancer cells were incubated for 48 h in the presence of test compounds [19,28,29]. Absorbances at 525 nm were measured in control wells (C) and test wells at time 0 (T_0_) and at the indicated times thereafter (T). Cell growth values were calculated as follows: (i) when T > T_0,_ cell growth (%) = 100 × ([T – T_0_]/[C – T_0_]), while (ii) when T < T_0_, cell growth (%) = 100 × ([T – T_0_]/T_0_). GI_50_ was calculated as 100 × ([T – T_0_]/[C – T_0_]) = 50. The LC_50_, an index of cytotoxic effect, was determined as the concentration of the compound at which 100 × (T – T_0_)/T_0_ = –50. The mean graph was produced by computer processing of the GI_50_ (LC_50_) values as described [19]. For each chemical, assays were performed using five concentrations (for example, 10^-4^, 10^-5^, 10^-6^, 10^-7^, and 10^-8^ M) and a negative control. All assays were performed in duplicate. Mean graphs, which show differential growth inhibition of each drug against the cell line panel, was generated based on calculations using a set of GI_50_ values [29,30]. To analyze correlations between the means of compounds A and B, we developed a COMPARE computer algorithm as described by Paull *et al.* [28]. Correlation coefficients were calculated according to the following formula: r = (Σ(x_i_-x_m_)(y_i_-y_m_))/(Σ(x_i_-x_m_)^2^Σ(y_i_-y_m_)^2^)^1/2^, in which x_i_ and y_i_ are log GI_50_ values for compounds A and Bagainst each cell line and x_m_ and y_m_ are the mean values of x_i_ and y_i_, respectively. We verified the accuracy of measured data by checking the dose response curves of reference control chemicals, such as mitomycin-C, paclitaxel, and SN-38, in every experiment.

### 3.3. Electrochemical measurements

Cyclic voltammetric measurements were performed on a conventional three-electrode system using a laboratory-constructed microcomputer-controlled system in which the working electrode potential was controlled by a potentiostat (Hokuto Denko, HA-301). Plastic-formed-carbon (PFC) electrodes with a surface area of 0.071 cm^2^ (BAS, PFCE-3), Ag/AgCl (saturated NaCl) electrodes, and platinum coil electrodes were used as the working, reference, and counter electrodes, respectively. Before recording each voltammogram, the working electrode was pretreated as previously described [13]. Aliquots of 0.05 mM heterocyclic quinone solutions in 3:1 (v/v) 0.1 M phosphate buffer containing 0.1 M KCl (pH 7.2)-ethanol were degassed using purified N_2_ gas prior to voltammetric measurements. The electrolytic cell was water-jacketed to maintain a constant temperature of 25 ± 0.1 ºC. 

### 3.4. Correlation coefficients

Correlations of the electrochemical and electronic parameters with the cytotoxic activities of heterocyclic quinones were determined using Pearson's correlation coefficient.

## 4. Conclusions

We have determined the growth inhibitory- and cytotoxic activities of 12 heterocyclic quinone anti-tumor agent candidates against a panel of 39 human cancer cell lines (JFCR39). The first reduction potentials, determined at a physiological pH (7.2), correlated with the cytotoxic activities against JFCR 39 of the heterocyclic quinones. In addition, log *P* and LUMO energy were also useful parameters to predict the cytotoxic activity. In our previous study, the first reduction potentials also correlated with the inhibitory effects of anthraquinone derivatives on EBV-EA activation; both the number of hydroxy groups and LUMO were useful parameters to predict inhibitory activity [18]. From these results, a reliable prediction of the cytotoxic activity of various quinone derivatives can be made using their reduction potentials without *in vitro* screening. 
